# Tuning ZnO-based piezoelectric nanogenerator efficiency through n-ZnO/p-NiO bulk interfacing

**DOI:** 10.1038/s41598-024-62789-3

**Published:** 2024-05-24

**Authors:** Abhinav Mahapatra, R. S. Ajimsha, Deepak Deepak, Pankaj Misra

**Affiliations:** 1 Oxide Nano-Electronics Lab, Laser Materials Processing Division, Raja Ramanna Centre for Advanced Technology, Indore, 452 013 India; 2 Homi Bhabha National Institute, Training School Complex, Anushakti Nagar, Mumbai, 400 085 India; 3 Department of Physics, School of Natural Sciences, Shiv Nadar Institution of Eminence (SNIoE), Greater Noida, 201314 Uttar Pradesh India

**Keywords:** Devices for energy harvesting, Semiconductors

## Abstract

ZnO based piezoelectric nanogenerators (PENG) hold immense potential for harvesting ambient vibrational mechanical energy into electrical energy, offering sustainable solutions in the field of self-powered sensors, wearable electronics, human–machine interactions etc. In this study, we have developed flexible ZnO-based PENGs by incorporating ZnO microparticles into PDMS matrix, with ZnO concentration ranging from 5 to 25 wt%. Among these, the PENG containing 15 wt% ZnO exhibited the best performance with an open-circuit output voltage/short-circuit current of ~ 42.4 V/2.4 µA. To further enhance the output performance of PENG, p-type NiO was interfaced with ZnO in a bulk hetero-junction geometry. The concentration of NiO was varied from 5 to 20 wt% with respect to ZnO and incorporated into the PDMS matrix to fabricate the PENGs. The PENG containing 10 wt% NiO exhibits the best performance with an open-circuit output voltage/short-circuit current of ~ 65 V/4.1 µA under loading conditions of 30 N and 4 Hz. The PENG exhibiting the best performance demonstrates a maximum instantaneous output power density ~ 37.9 µW/cm^2^ across a load resistance of 20 MΩ under loading conditions of 30 N and 4 Hz, with a power density per unit force and Hertz of about ~ 0.32 µW/cm^2^·N·Hz. The enhanced output performance of the PENG is attributed to the reduction in free electron concentration, which suppresses the internal screening effect of the piezopotential. To assess the practical utility of the optimized PENG, we tested the powering capability by charging various commercial capacitors and used the stored energy to illuminate 10 LEDs and to power a stopwatch displays. This work not only presents a straightforward, cost-effective, and scalable technique for enhancing the output performance of ZnO-based PENGs but also sheds light on its underlying mechanism.

## Introduction

Piezoelectric nanogenerators (PENG) have attracted significant attention owing to the advancements in low power electronics and the increasing demand for portable devices. PENGs have emerged as a potential candidate in many self-powered applications, such as self-powered UV detection, gas sensing, biomedical devices, footstep counting, and environmental monitoring^1–6^. In addition, the need for frequent replacing/recharging and disposal issue of bulky batteries used in sensors has prompted researchers to explore self-powering alternatives.

Several materials have been investigated for the fabrication of PENGs such as BaTiO_3_, MoS_2_, ZnO, PVDF, PZT, LiNbO_3_, AlN, KNbO_3_ and PMN-PT etc.^7–16^. Among them ZnO has been chosen due to its lead-free, semiconducting, biocompatible, low-cost and well established growth techniques. However, the screening of piezoelectric voltage due to the presence of free electrons in ZnO limits its effective utilization in the fabrication of PENG^17,18^. Due to the presence of oxygen vacancies and zinc interstitials ZnO act like a n-type semiconductor. Many approaches have been opted to neutralize the excess electrons in ZnO which screens the piezoelectric potential such as doping, plasma treatment, annealing, fabrication of thin film based p–n junction and bulk interfacing of p and n type of material etc.^9,19–23^.The utilization of plasma treatment, annealing and doping techniques in enhancing the output characteristics of PENGs is hindered by the reversible effects induced by plasma treatment or annealing^24^ and the reliance on toxic and expensive dopants.

The fabrication of heterojunctions and p-n junctions holds significant technological importance for enhancing performance of semiconductor in various devices, such as semiconductor lasers, LEDs, photodetectors, and solar cells^25^. There are reports based on p–n junction heterojunction in PENGs to reduce the free electron induced screening of piezopotential and thereby enhancing its output characteristics, though extensive studies in this direction are scarcely available. Similar works were reported in the case of ZnO by interfacing with p-type organic and inorganic materials such as Spiro-MeOTAD, P3HT:PCBM, PEDOT:PSS, Cu_2_O, CuO, NiO and CuI etc.^21,23,26–30^. The organic p-type materials have demonstrated enhanced charge transfer properties but their practical implementation is hindered by there inherent instability, high cost, susceptibility to contamination, and large band gaps^31,32^. In order to circumvent these issues, interfacing ZnO with more stable and cost-effective inorganic p-type semiconductors emerged as a plausible alternative. Conventionally, thin-film based p-n junctions were fabricated using expensive deposition techniques such as atomic layer deposition, sputtering, pulsed layer deposition, and metal organic chemical vapor deposition. However, the bulk interfacing of p and n materials facilitates intimate mixing, thereby enhancing the interface area by formation of numerous p-n interfaces within a single layer^33^. The bulk-interfaced p–n junction can be deposited via simple synthesis routes on large-area flexible substrates at low temperature^34^, thereby reducing fabrication costs and enhancing scalability. Therefore, we have adopted this straightforward, cost-effective, and scalable approach involving the bulk interfacing of inorganic p-type NiO and n-type ZnO to ameliorate the output characteristics of PENGs.

In this work, p-type NiO was chosen to form bulk heterojunction with ZnO due to its cost-effectiveness and high hole concentration. The PENG with 15 wt% concentration of ZnO in PDMS demonstrated the best output performance with open-circuit output voltage/short-circuit current values of ~ 42.4 V/2.4 µA. To further enhance the output performance of PENG, various bulk heterojunctions of NiO and 15 wt% concentration of ZnO in PDMS were fabricated. Among them the PENG with 10 wt% NiO concentration exhibits best output performance with open-circuit output voltage/short-circuit current values ~ 65 V/ 4.1 µA. The enhancement in output performance of PENG was due to the reduced screening effect of piezopotential due to free electrons. Powering capability of the fabricated PENG was demonstrated by charging various commercial capacitors and finally the stored energy in capacitor was used to power 10 LEDs and stopwatch display.

## Experimental section

The high purity (99.9995%) ZnO and NiO to fabricate the PENGs. ZnO was annealed at 550 ºC for 3 h in oxygen ambient, since it is already reported that PENG fabricated using annealed ZnO shows higher output as compared to non-annealed ZnO^9^. Annealed ZnO was finely grounded for 30 min using mortar pestle to avoid agglomeration. NiO was grounded with ZnO using mortar pestle to interface n-type ZnO and p-type NiO in bulk form. To fabricate ZnO- PENG, ZnO with different concentrations from 5 to 25 wt% was incorporated into PDMS matrix thoroughly using continuous magnetic stirring for 30 min. The prepared ZnO:PDMS composite was spin coated at 650 rpm for 45 s over the flexible ITO coated PET substrate. The deposited ZnO:PDMS composites were dried at 75 °C for 3 h inside oven. The final PENG devices were fabricated with sandwich device configuration PET/ITO/ZnO:PDMS/ITO/PET, which was realized by mechanically integrating the ITO coated PET over ZnO:PDMS composite surface. In case of ZnO-NiO based PENG the entire procedure of fabrication is same with NiO concentration varied from 5 to 20 wt% with respect to ZnO. The final connections were taken from the top and bottom electrodes of the device.

The ZnO and NiO structural analysis was conducted via X-ray diffraction, utilizing Cu-Kα radiation with a wavelength of 1.5405 Å. The morphological details were investigated using a field emission scanning electron microscope (FESEM) with a JEOL JSM-7610 F. Vibrational modes were investigated using Raman spectroscopy with a micro-Raman spectrometer (STR) fitted with a 532 nm argon-ion laser source operating at 2.5 mW power and a 50X magnification objective lens. Photoluminescence measurements were carried out using a 30 mW He–Cd laser operating at 325 nm as an excitation source. Luminescence data were collected and detected using a spectrometer (Triax 550, Jobin Yvon, France) connected to a CCD detector (Andor, UK). Electrical measurements were carried out utilizing a Keithley 2636B source measure unit. Furthermore, the characterization of the piezoelectric nanogenerator was performed using a linear motor with controlled force and frequency. The output characteristics of the piezoelectric nanogenerator were measured across a Lecroy oscilloscope (Waverunner 8104) and a Stanford low-noise current pre-amplifier (SRS-SR570) for voltage and current measurement, respectively.

## Results and discussions

**Figure 1:**
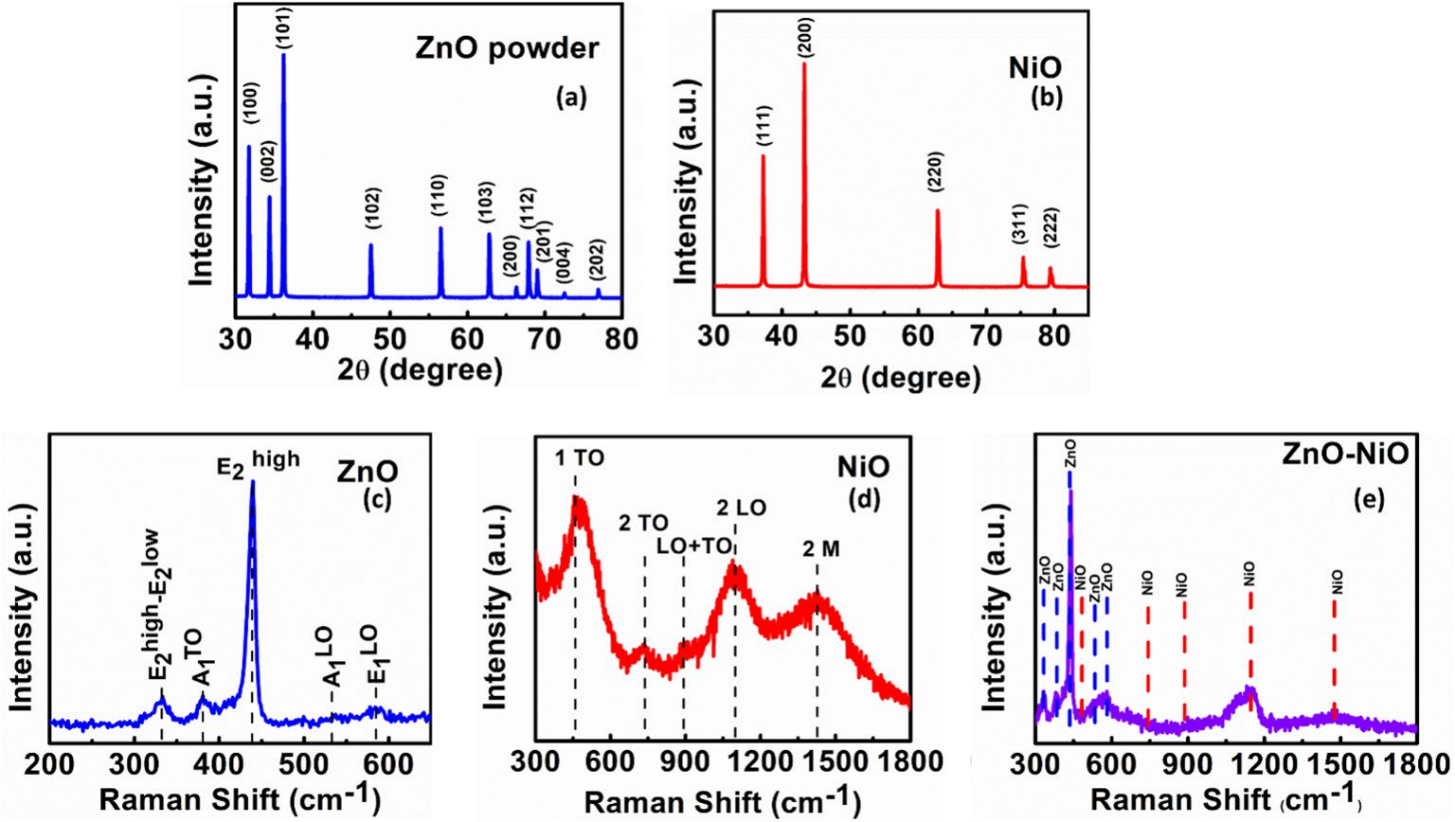
X-ray diffraction pattern of (**a**) ZnO and (**b**) NiO and Raman Spectra of (**c**) ZnO, (**d**) NiO, (**e**) ZnO-NiO interfaced sample.

Figure 1a shows the x-ray diffraction pattern (XRD) of ZnO which exhibits hexagonal wurtzite structure of ZnO (P63mc)^35^. Figure 1b illustrates the XRD pattern of NiO exhibiting cubic rock salt crystal structure with space group (Fm-3 m)^36^. Both the materials show sharp characteristics peak which exhibits the high crystallinity of the materials.

Figure 1c–e shows the Raman spectra of ZnO, NiO and ZnO-NiO interfaced samples, respectively. The Raman spectra of ZnO shows five Raman peaks at 330 cm^−1^, 382 cm^−1^, 438 cm^−1^, 530 cm^−1^ and 580 cm^−1^. The peak at 330 cm^−1^ shows multiphonon progression associated with E_2_^high^–E_2_^low^ second-order vibration mode^37^. The peaks at 382 cm^−1^ and 438 cm^−1^ represent the A_1_^TO^ mode and the E_2_^high^ optical mode vibrations respectively which show the hexagonal wurtzite phase of ZnO with high crystallinity^38^. The two minor peaks at 530 cm^−1^ and 580 cm^−1^ were assigned to A_1_^LO^ and E_1_^LO^ vibrational modes^39^.The Raman spectra of NiO exhibit five distinct combinations of longitudinal optical (LO), transverse optical (TO), and magnon vibrational modes detected at frequencies of 476 cm^−1^, 724 cm^−1^, 904 cm^−1^, 1087 cm^−1^, and 1419 cm^−1^^40^. These modes correspond to the one-phonon 1 TO mode, the two-phonon 2 TO mode, the combination of LO and TO vibrational modes (LO + TO), two LO mode vibrational modes, and a two-magnon mode (2 M)^40–42^.The Raman spectra of the ZnO-NiO interfaced sample exhibits peaks that corresponds to ZnO and NiO, eliminates the possibility of Ni doping within ZnO or change in the chemical composition. These findings suggest the formation of possible interface between n-type ZnO and p-type NiO which leads to the neutralization of excess electrons in ZnO, resulting in a lowering of screening effect tending to the enhancement in the piezoelectric potential.

**Figure 2:**
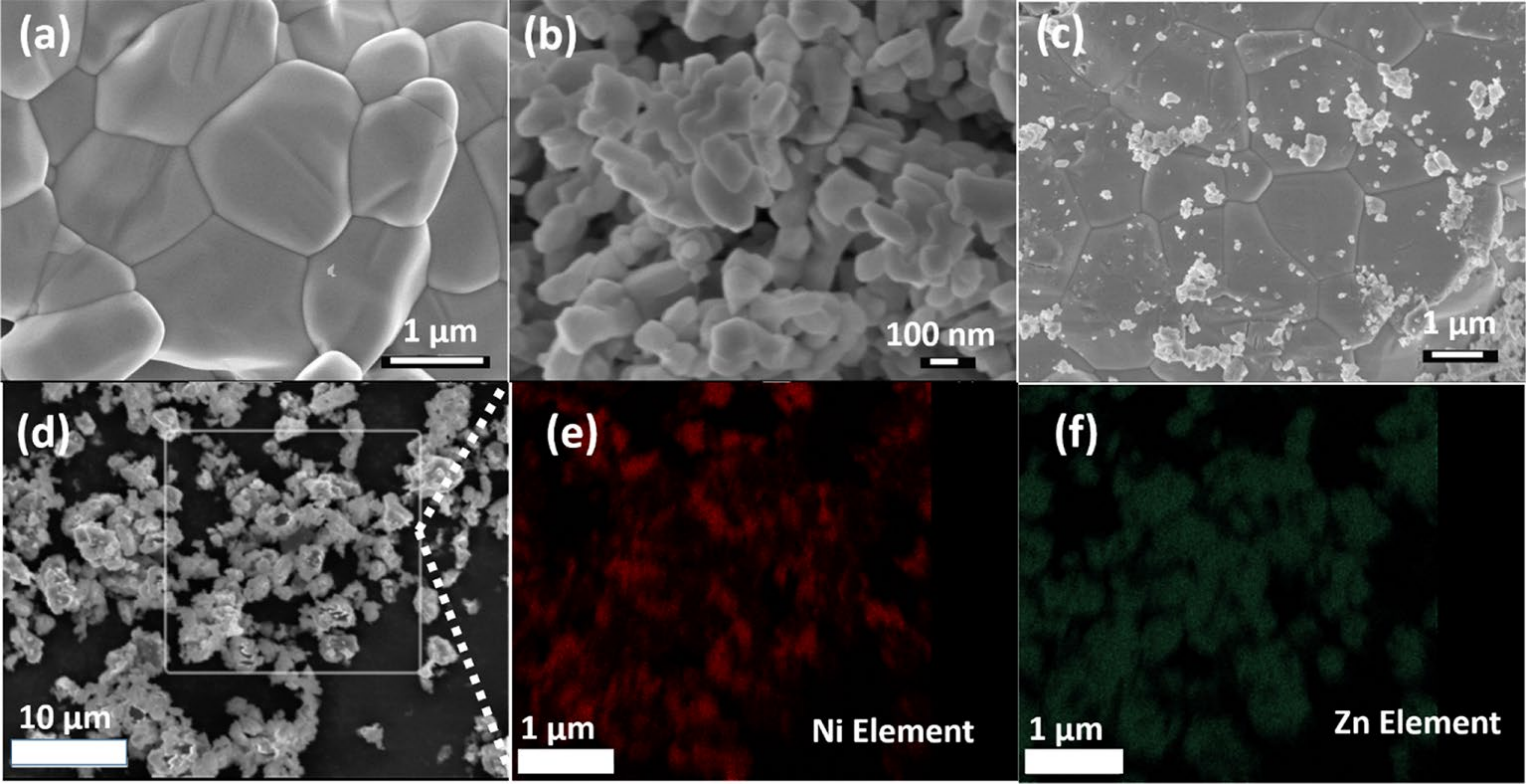
FESEM image of (**a**) ZnO (**b**) NiO and (**c**) ZnO-NiO interfaced sample. (**d**) Area scanned for elemental analysis of ZnO-NiO interfaced sample (**e**) elemental map Ni element and (**f**) Zn element.

Figure 2a shows the SEM image of annealed ZnO microparticles, with particle sizes ranging from 1 to 5 µm. The size of ZnO microparticles were advantageous for establishing multiple interfaces with NiO nanoparticles, given the nano-dimensions of NiO. The rice-like morphology of the NiO nanoparticles is shown in Fig. 2b. Figure 2c displays the SEM image of the ZnO-NiO interfaced sample, illustrating the formation of multiple interfaces resulting from the grinding of NiO nanoparticles with ZnO microparticles, where the smaller NiO nanoparticles adhere to the larger ZnO microparticles. Figure S2a in supplementary material shows the statistical distribution of size of ZnO-NiO bulk heterojunction particles ranging from 1.26 to 6.035 µm, with an average particle size of 2.14 µm. Figure S2b in supplementary material shows the cross-sectional image of ZnO_15_/NiO_10_:PDMS which shows the uniform distribution of particles inside the PDMS matrix. To verify the chemical composition of these nanoparticles, an elemental analysis was performed on an area of the ZnO-NiO interfaced sample, as shown in Fig. 2d. Figure 2e,f exhibits the elemental maps of Ni and Zn, respectively, confirming the presence of NiO and ZnO in the sample. The elemental mapping clearly indicates that the nanoparticles correspond to NiO, whereas the microparticles correspond to ZnO. Therefore, the SEM images and elemental analysis confirm the formation of multiple interfaces between NiO nanoparticles and ZnO microparticles. Figure S3 of supplementary material shows the EDS spectra of ZnO/NiO heterojunction inside PDMS, presence of both the elements implies the integrity of heterojunction inside PDMS.

**Figure 3:**
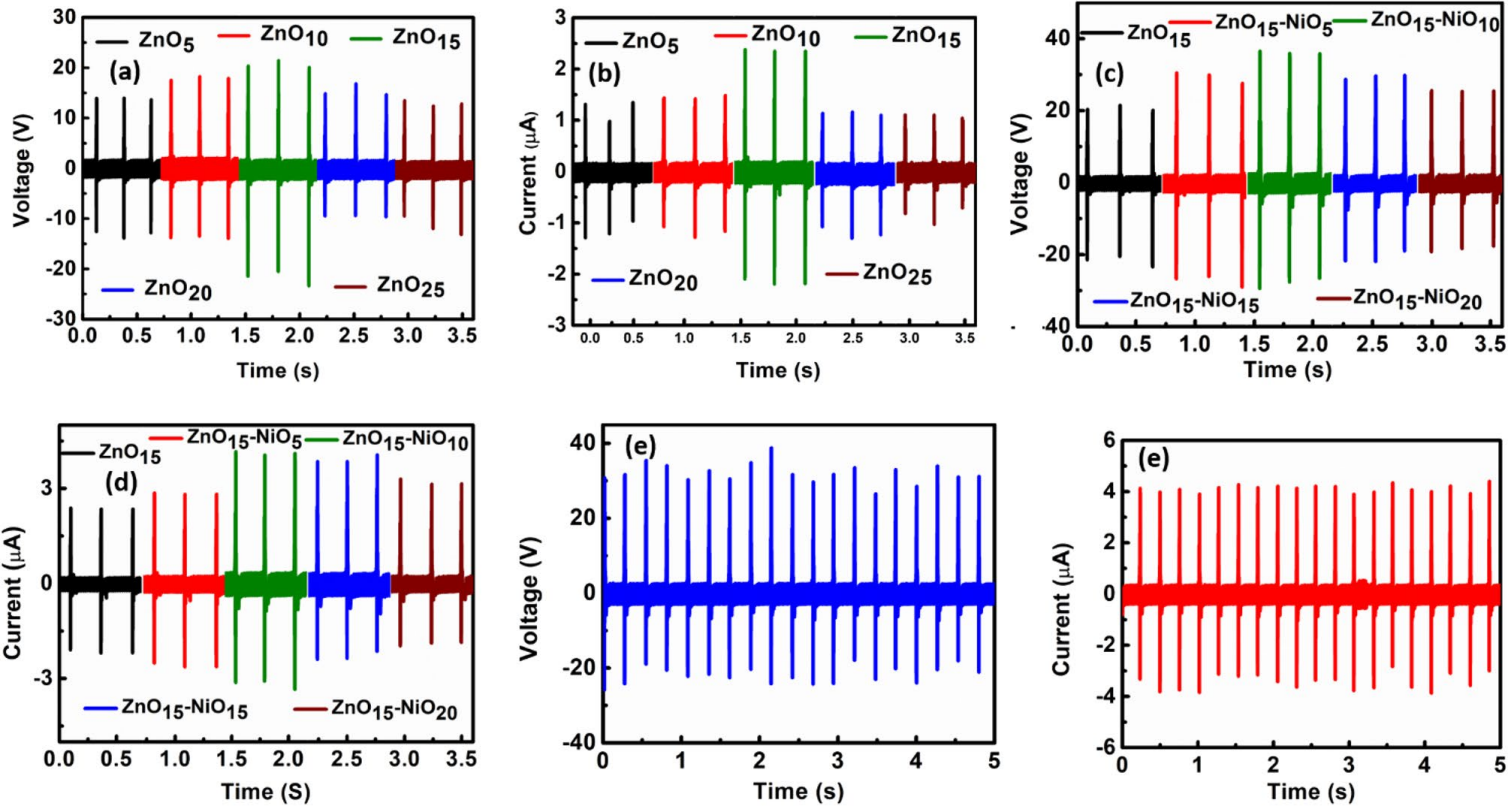
(**a**) Open-circuit output voltage and (**b**) short-circuit output current variation with concentration of ZnO in PDMS matrix, (**c**) open-circuit output voltage and (**d**) short-circuit output current variation with concentration of NiO with respect to ZnO, (**e**) Open-circuit output voltage and (**f**) short-circuit output current of PENG with best performance (ZnO_15_-NiO_5_:PDMS).

Piezoelectric nanogenerators (PENG) working principle is based on the direct piezoelectric effect, generating voltage across a material when force is applied. Initially, we optimized the ZnO concentration in PDMS by varying it from 5 to 25 wt% relative to PDMS. Subsequently, PENGs were fabricated using these compositions and subjected to a continuous force of 30 N at a frequency of 4 Hz for testing. As shown in Fig. 3a,b, the open-circuit output voltage/short-circuit current of the PENGs exhibited an increase from ~ 27 V/1.32 µA to ~ 42.4 V/2.4 µA with the increase in ZnO wt% up to 15 wt%. However, the output characteristics decreased to ~ 24.5 V/1.17 µA and ~ 23 V/1.15 µA for 20 wt% and 25 wt%, respectively. The observed enhancement in output characteristics with increased ZnO concentration correlates with the higher concentration of piezoelectric ZnO in the PDMS matrix. The decline in output performance beyond 15 wt% is attributed to Maxwell–Wagner interfacial polarization due to the presence of PDMS and ZnO as two different phases in the composite^9^. The higher concentration of ZnO within the PDMS matrix leads to an increased number of interfaces with PDMS, promoting Maxwell–Wagner interfacial polarization, consequently enhancing the overall dielectric constant of the composite^43^. Equation (1)^44^ shows the output voltage (V) of piezoelectric nanogenerator, Where, d is the piezoelectric coefficient, ε_0_ is the permittivity of free space and ε_r_ is the relative permittivity, F is the external force on the device, t is the thickness of the piezoelectric composite and A is the area of the device.

1 $$V=\frac{dFt}{{\varepsilon }_{0}{\varepsilon }_{r}A}.$$

The piezoelectric output voltage is directly proportional to the ZnO concentration but inversely proportional to the dielectric constant of composite. The abrupt increase in dielectric constant at higher ZnO concentrations due to the Maxwell–Wagner effect contribution is responsible for the deterioration in output performance beyond 15 wt%.

**Figure 4:**
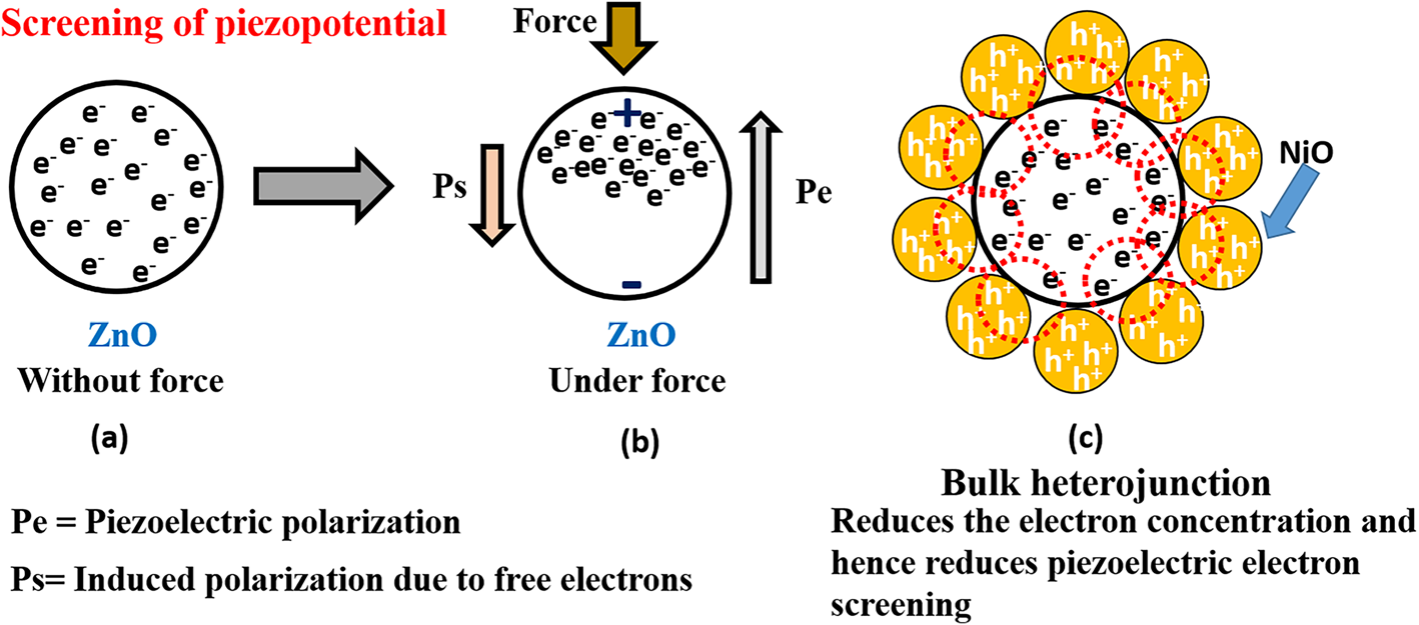
(**a**) ZnO without any force, (**b**) partial polarization of free electrons due to piezopotential and (**c**) reduction of free electron concentration due to the formation of bulk heterojunction.

To enhance the output performance of the PENG, we have interfaced p-type NiO with n-ZnO with specific interest of reducing the free electron concentration in ZnO. We have simply grinded the NiO in different concentration ranging from 5 to 20 wt% with respect to optimized ZnO concentration in PDMS. The grinded interfaced ZnO-NiO is then incorporated into PDMS matrix to fabricate PENGs. The PENGs were tested under the same conditions of force and frequency i.e. 30 N and 4 Hz. The open-circuit output voltage/short-circuit current of the PENG enhanced from ~ 42.4 V/2.4 µA to ~ 65 V/4.1 µA with increase in concentration of NiO with respect to ZnO from 0 wt% to 10 wt% (as shown in Fig. 3c,d). With further increase in concentration of NiO the output of the PENG decreased to ~ 51 V/3.9 µA and ~ 44 V/3.2 µA for 15 wt% and 20 wt%, respectively. In Fig. 4a, the screening of the piezoelectric effect due to free electrons is illustrated. As shown in Fig. 4b, when a force is applied to the ZnO material, a voltage develops across it due to the direct piezoelectric effect. Consequently, the free electrons within the material migrate towards the positive potential side, inducing a polarization opposite to the piezoelectric potential generated by the force application. As shown in Fig. 4c, to mitigate the presence of excess electrons, multiple interfaces of n-ZnO/p-NiO have been fabricated through bulk interfacing. These interfaces effectively deplete the electron concentration near the junction area, thereby reducing the overall electron concentration within the ZnO material. Consequently, the screening of the piezoelectric potential is also diminished due to the reduction in electron concentration. To confirm the contribution of PDMS and NiO to the piezoelectric output, we fabricated two PENGs: one with PDMS and the other with NiO:PDMS in a sandwiched structure. We have performed measurements on both PENGs. Figure S1 in the supplementary material displays the output voltage/output current of both PENGs, which were ~ 0.2 mV/0.1 nA. This output is negligible compared to the output of PENGs containing ZnO. This suggests that the piezoelectric output signal generated by the PENG is solely attributed to ZnO. To investigate the reason of enhancement in output performance with NiO interfacing and validate the explanation the explanation, room temperature photoluminescence and I-V measurements has been carried out on the pelletized samples of ZnO and ZnO-NiO_10_. Photoluminescence and I–V measurements has been carried out on the pelletized samples of ZnO and ZnO-NiO_10_.

**Figure 5:**
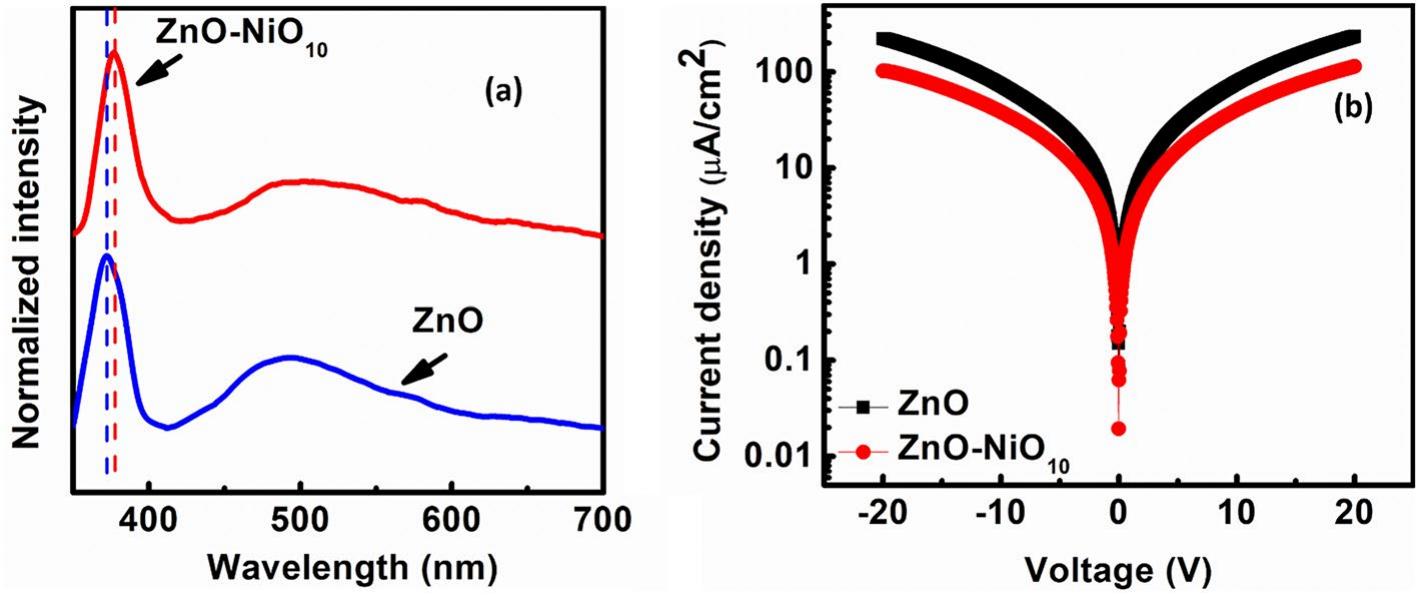
(**a**) Photoluminescence spectra and (**b**) I-V measurements of ZnO and ZnO-NiO_10_ pelletized sample.

**Figure 6:**
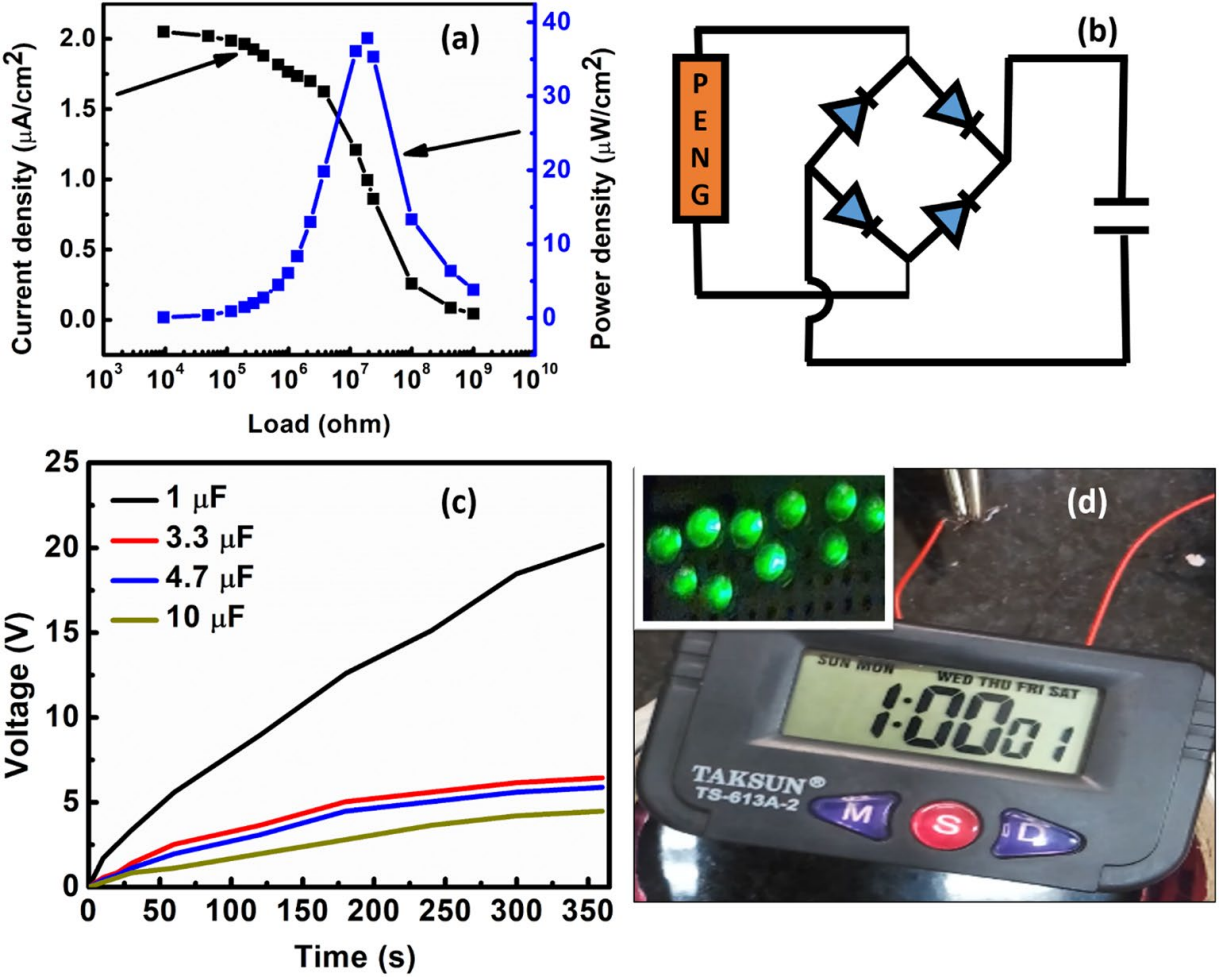
(**a**) Load matching analysis on optimized PENG. (**b**) Circuit used to charge capacitors. (**c**) Charging of commercial capacitors using rectified output of PENG. (**d**) Flashing of LEDs and stopwatch display using the stored energy in capacitors.

Figure 5a shows the photoluminescence spectra of ZnO and ZnO-NiO_10_ interfaced samples. The strong characteristic near band edge emission (NBE) peak for ZnO was observed at ~ 372 nm (3.33 eV) is red shifted to ~ 376 nm (3.29 eV) for ZnO-NiO_10_ interfaced samples. This observed red shift is attributed to the Burstein Moss (BM) shift, signifying a decrease in carrier concentration upon interfacing with NiO^45^. In addition to NBE, both the samples exhibit midgap luminescence originates from intrinsic defects. ZnO and ZnO:NiO_10_ samples shows luminescence peak at ~ 498 nm (~ 2.5 eV), which can be attributed to originated from oxygen vacancies defects^45,46^. Oxygen vacancies generally act as electron donor defect to increase the carrier concentration in ZnO. With NiO interfacing, the intensity of the oxygen vacancy peak is decreased implying a reduction in electron-donating oxygen vacancies and consequently a decline in electron concentration within the ZnO. Figure 5b shows the semi-log plot of current density versus applied voltage (J–V) for ZnO and ZnO-NiO_10_ interfaced samples. The current density of the ZnO is decreased from 234.4 to 113 µA/cm^2^ with NiO interfacing, which further confirms the reduction of carrier concentration in ZnO with NiO interfacing. So, it is concluded that the enhancement in output performance of the PENG with NiO interfacing is due to the reduction in electron concentration in ZnO which is responsible for the screening of piezoelectric potential due to carrier concentration. However, the decline in output performance when incorporating NiO beyond 10 wt% might be attributed to an excessive presence of holes in the composite material. When an external force is applied to the PENG, the piezoelectric potential developed within the material. However, the surplus of holes induces an opposing space charge polarization inside the material, thereby further screening the piezoelectric potential and subsequently reducing the output performance. Hence, the interplay between electron and hole concentrations is crucial for minimizing the screening of the piezoelectric potential. Current across various loads connected to the PENG was measured in order to determine the instantaneous output power of optimized PENG. Equation (2) was used to evaluate the output power density of the PENG:

2 $$P=\frac{{I}^{2}R}{A}$$

where I represent the current measured across various load resistances R, and A denotes the area of the PENG device. The variation of output current and output power density with externally connected load is shown in Fig. 6a. When the connected load across the PENG was varied from 10 kΩ to 1 GΩ, the current density decreased from 2.05 to 0.04 µA/cm^2^. The maximum instantaneous output power density of ~ 37.9 µW/cm^2^ was observed across a load resistance of 20 MΩ under loading conditions of 30 N and 4 Hz. The power density/ (Force × Hz) under the mentioned loading condition is ~ 0.32 µW/cm^2^ N Hz, which is consistent with earlier reported results^46^. To demonstrate the powering ability of the optimized PENG the output of the PENG was rectified using full wave rectifier IC (W04M). As shown in the circuit of Fig. 6b, the output of the PENG was coupled directly to the input of the rectifier IC, and the output of the IC was connected to the commercial capacitors. Figure 6c shows the charging of commercial capacitors of 1 µF, 3.3 µF, 4.7 µF and 10 µF using rectified output of the PENG. The capacitor of 1 µF stored the maximum voltage of ~ 20.1 V in 6 min of tapping. The stored energy was used directly to flash 10 commercial LEDs and to power a stopwatch display as shown in Fig. 6d. Thus, the proposed bulk interfacing of p-NiO and n-ZnO offers a straightforward, cost-effective, and scalable technique to enhance the output performance of ZnO-based PENG. This is achieved by annulment of excess electrons, thereby reducing the screening effect of the piezoelectric potential. Moreover, the optimized PENG demonstrates the capability of harvesting vibrational energy to power LEDs and a stopwatch display.

## Conclusion

ZnO:PDMS based PENGs were fabricated by varying the concentration of ZnO from 5 wt% to 25 wt%. PENG with 10 wt% of ZnO shows the best output performance with open-circuit output voltage/short-circuit current of ~ 42.4 V/2.4 µA. The output performance of the ZnO:PDMS based PENG was improved by a straightforward, cost-effective, and scalable technique of bulk interfacing with p-type NiO. The PENG with 10 wt% of NiO with respect of ZnO exhibits the best enhanced output characteristics of ~ 65 V/4.1 µA with a maximum instantaneous output power of ~ 37.9 µW/cm^2^. The enhancement in output performance is due to the annulation of free charge carrier responsible for screening of generated piezoelectric potential. The rectified output of the PENG was used to charge various commercial capacitors. The capacitor of 1 µF stored the maximum voltage of ~ 20.1 V. The stored energy was used to power 10 commercial LEDs and stopwatch display.

### Supplementary Information

Supplementary Information.

## Data Availability

The data that supports the findings of this study are available within the article.
