# Supplementary material for: Tuning ZnO-based piezoelectric nanogenerator efficiency through n-ZnO/p-NiO bulk interfacing
Source: Sci Rep. 2024 May 24;14:11871. doi: 10.1038/s41598-024-62789-3 (PMC11126727; doi:10.1038/s41598-024-62789-3)
Supplement: Supplementary file 1 — Supplementary Information. [file 41598_2024_62789_MOESM1_ESM.pdf]

Supporting Information

Tuning ZnO-based piezoelectric nanogenerator efficiency through n-ZnO/p-NiO bulk interfacing

Abhinav Mahapatra [a, b]*, R.S. Ajimsha [a], Deepak Deepak [c], Pankaj Misra [a, b]

[a] *Oxide Nano-Electronics Lab, Laser Materials Processing Division, Raja Ramanna Centre for Advanced Technology, Indore 452 013, India*

[b] *Homi Bhabha National Institute, Training School Complex, Anushakti Nagar, Mumbai 400 085, India*

[c] *Department of Physics, School of Natural Sciences, Shiv Nadar Institution of Eminence (SNIoE), Greater Noida, Uttar Pradesh 201314, India*

[*] Corresponding author Email: patraabhi007@gmail.com (A. Mahapatra) , pmisra@rrcat.gov.in (P. Misra)

---

1. Output signals from PDMS and NiO:PDMS under periodic external force

Figure S1 (a,b) and (c,d) shows the output characteristics of PDMS and NiO:PDMS, respectively, under a constant periodic external force of 30 N at 4 Hz, exhibiting an output voltage/output current of approximately 0.2 mV/0.1 nA. These observed characteristics are negligible compared to the output of fabricated PENGs with ZnO. This suggests that the piezoelectric output signal generated from the PENG is solely attributed to ZnO.

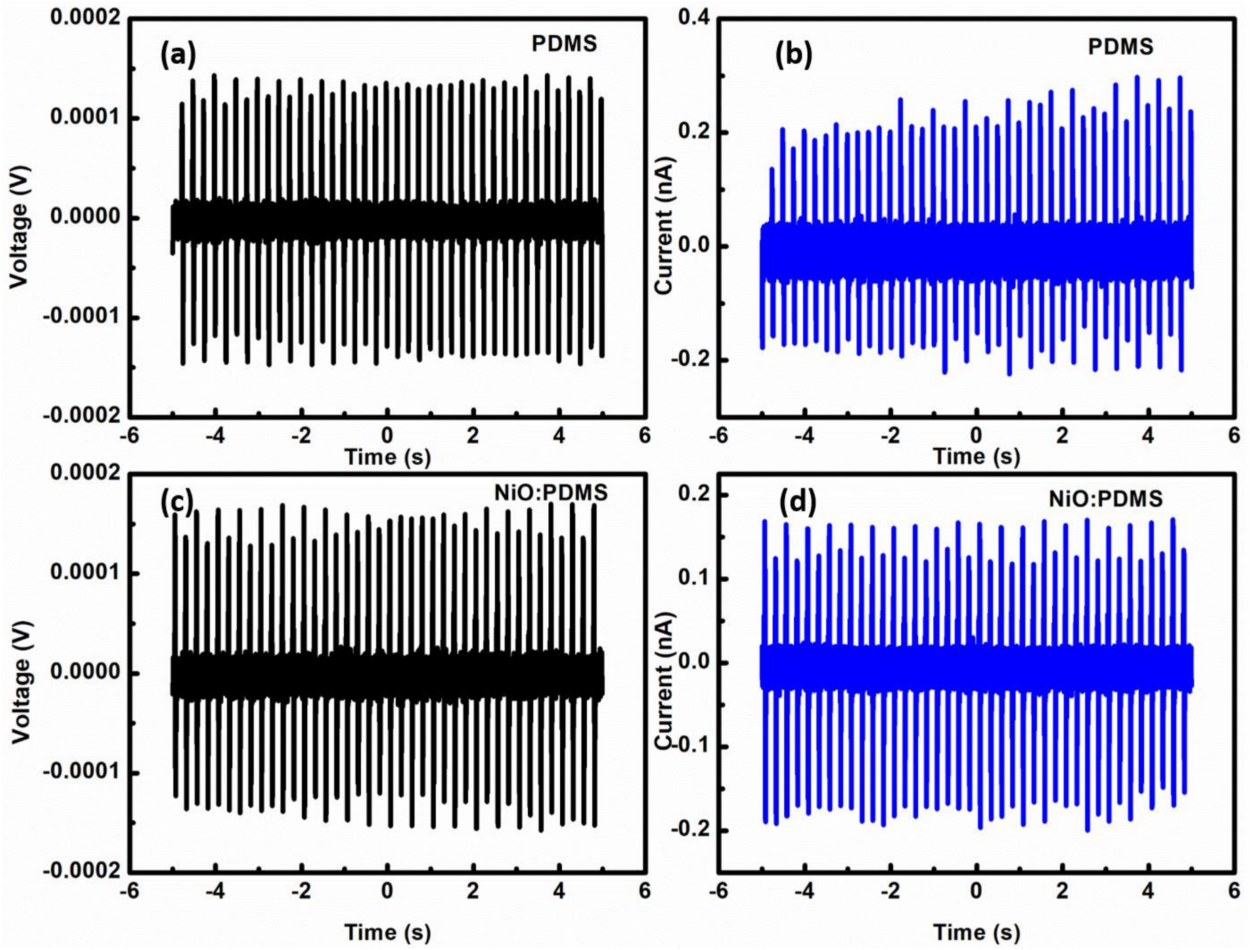

Figure S1: (a) Output voltage and **(b)** current of PDMS based PENG**, (c)** output voltage and **(d)** current of NiO:PDMS based PENG

2. Statistical analysis of particle size of ZnO/NiO bulk heterojunction and cross sectional image ZnO/NiO PDMS composite

Figure S2 (a) shows the FESEM image of the ZnO/NiO bulk heterojunction. We have carried out a size distribution analysis of the ZnO particles ranging from 1.26 to 6.035 µm, with an average particle size of 2.14 µm. In Figure S2 (b), the cross-sectional image of the ZnO/NiO:PDMS composite illustrates the uniform distribution of particles within the PDMS matrix.

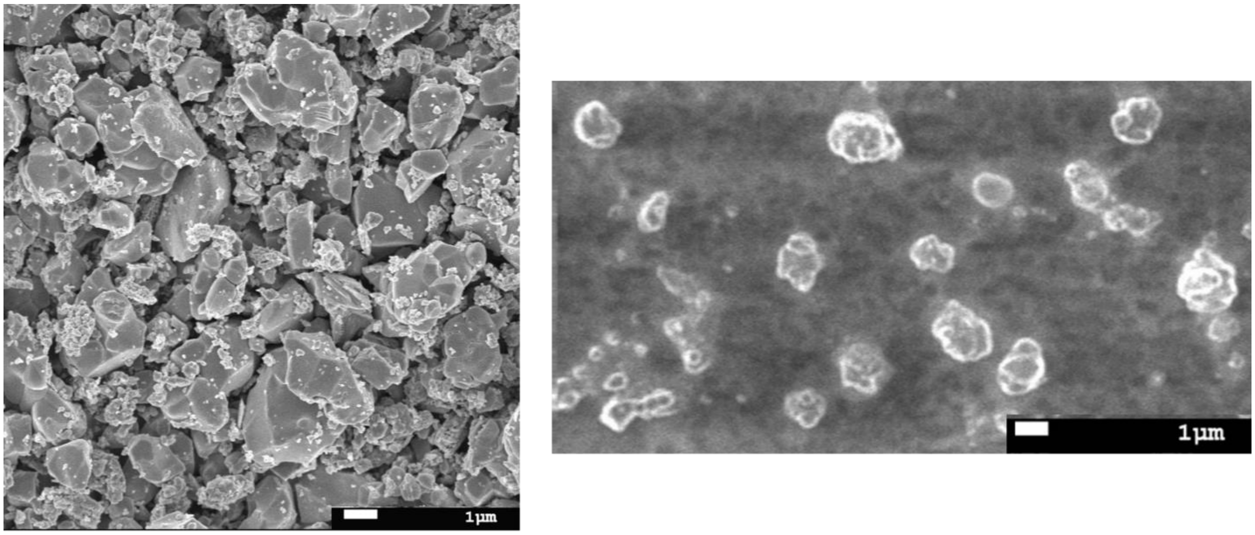

(a) (b)

Figure S2: (a) FESEM image of ZnO/NiO bulk heterojunction **(b)** cross-sectional FESEM image of ZnO/NiO:PDMS composite

3. EDS plot of ZnO/NiO heterojunction inside PDMS

As shown in Figure S3, we conducted EDS measurements on a cross-sectional SEM image of the ZnO/NiO:PDMS composite. The EDS analysis exhibits peaks corresponding to both Zn and Ni, indicating the presence of ZnO and NiO within the composite. This implies that the ZnO/NiO bulk heterojunction remains intact within the PDMS matrix.

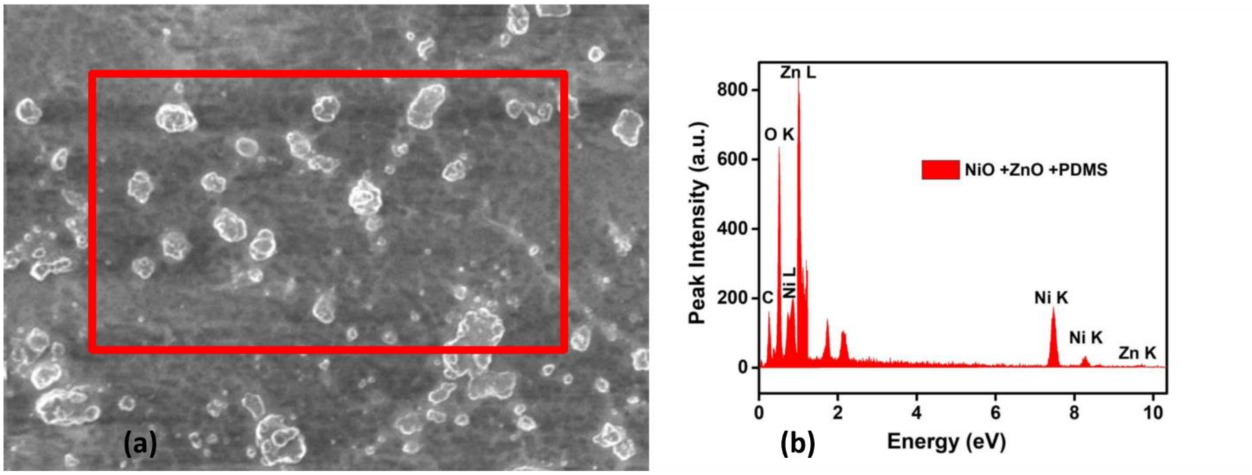

eZAF Smart Quant Results

Element	Weight %	Atomic %	Net Int.	Error %	Kratio	Z	A	F
C K	8.03	10.14	18.34	72.19	0.0331	0.8258	0.5925	1.0000
O K	15.33	24.23	91.20	5.46	0.0933	0.7732	0.7870	1.0000
NiK	13.82	10.95	52.49	6.45	0.4609	0.7353	1.0029	1.0021
ZnK	62.82	54.68	1.29	10.44	0.0193	0.7467	0.9919	1.0068

Figure S3: (a) FESEM image of ZnO/NiO:PDMS, on which EDS measurements were conducted; **(b)** EDS spectra of the composite, displaying the elemental compositions

4. Zoomed piezoelectric voltage signal of best PENG

The voltage signal from the PENG consists of two parts: the main piezoelectric signal and another signal attributed to damped remnant vibrations following the removal of the applied force.

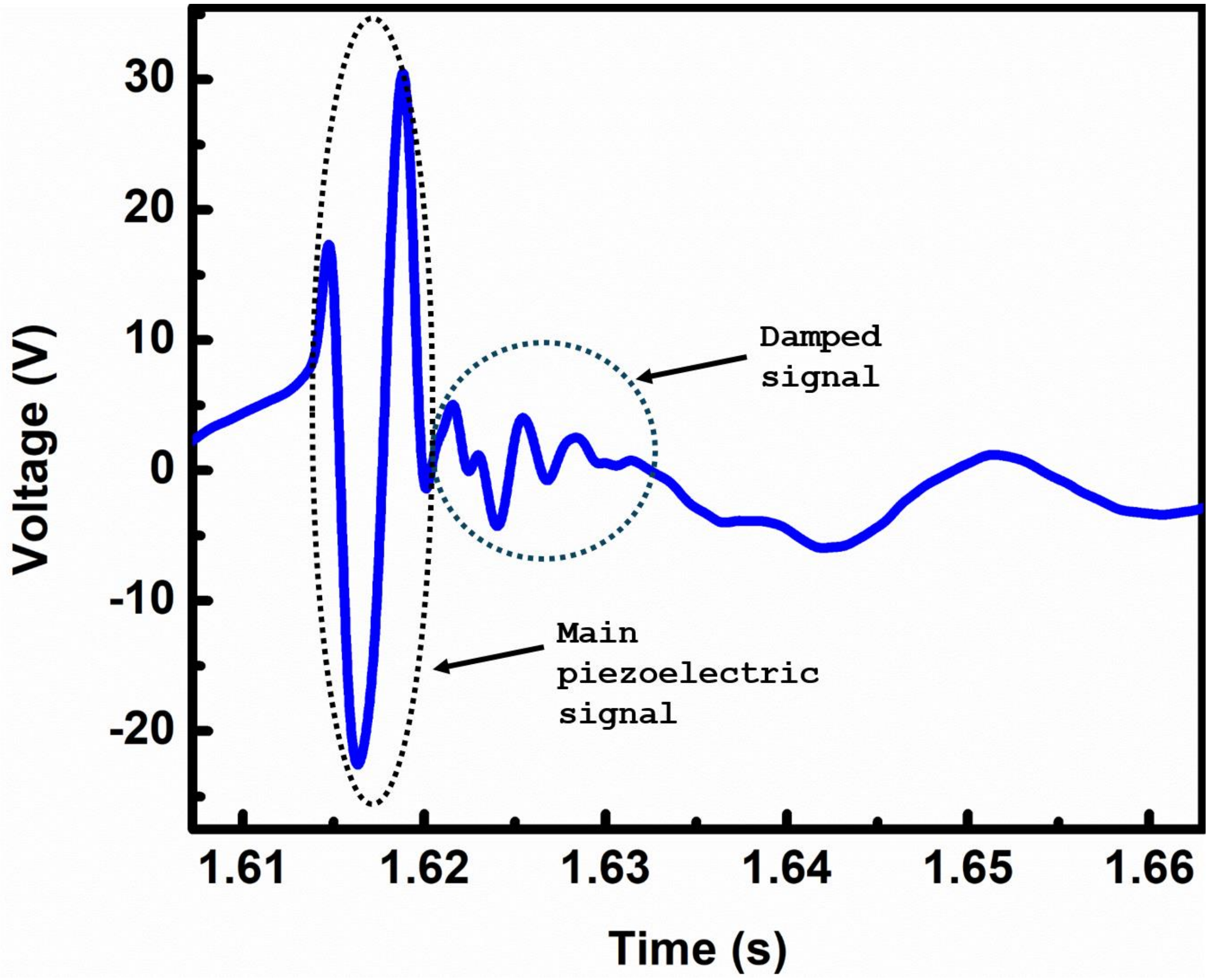

Figure S4: The zoomed piezoelectric voltage of ZnO_{15}/NiO_{10}:PDMS based PENG
